# The Effect of Some Fluoroquinolone Family Members on Biospeciation of Copper(II), Nickel(II) and Zinc(II) Ions in Human Plasma

**DOI:** 10.3390/molecules190812194

**Published:** 2014-08-13

**Authors:** Predrag Djurdjevic, Ivan Jakovljevic, Ljubinka Joksovic, Nevena Ivanovic, Milena Jelikic-Stankov

**Affiliations:** 1 Faculty of Science, Chemistry Department, University of Kragujevac, P.O.BOX 60, 34000 Kragujevac, Serbia; E-Mails: ivan_jakovljevic@kg.ac.rs (I.J.); ljubinka@kg.ac.rs (L.J.); n.ivanovic@kg.ac.rs (N.I.); 2 Faculty of Pharmacy, Analytical Chemistry Department, University of Belgrade, 11221 Belgrade, Serbia; E-Mail: mjstankov@pharmacy.bg.ac.rs

**Keywords:** copper, nickel, zinc, fluoroquinolones, complexes, biospeciation, blood plasma, computer simulation

## Abstract

The speciation of Cu^2+^, Ni^2+^ and Zn^2+^ ions in the presence of the fluoroquinolones (FQs) moxifloxacin, ofloxacin, levofloxacin and ciprofloxacin, in human blood plasma was studied under physiological conditions by computer simulation. The speciation was calculated using an updated model of human blood plasma including over 6,000 species with the aid of the program Hyss2009. The identity and stability of metal-FQ complexes were determined by potentiometric (310 K, 0.15 mol/L NaCl), spectrophotometric, spectrofluorimetric, ESI-MS and ^1^H-NMR measurements. In the case of Cu^2+^ ion the concentration of main low molecular weight (LMW) plasma complex (Cu(Cis)His) is very slightly influenced by all examined FQs. FQs show much higher influence on main plasma Ni^2+^ and Zn^2+^ complexes: (Ni(His)_2_ and Zn(Cys)Cit, respectively. Levofloxacin exhibits the highest influence on the fraction of the main nickel complex, Ni(His)_2_, even at a concentration level of 3 × 10^−5^ mol/L. The same effect is seen on the main zinc complex, Zn(Cys)Cit. Calculated plasma mobilizing indexes indicate that ciprofloxacin possesses the highest mobilizing power from plasma proteins, toward copper ion, while levofloxacin is the most influential on nickel and zinc ions. The results obtained indicate that the drugs studied are safe in relation to mobilization of essential metal ions under physiological conditions. The observed effects were explained in terms of competitive equilibrium reactions between the FQs and the main LMW complexes of the metal ions.

## 1. Introduction

Elemental speciation in biological fluids, particularly human plasma, implies investigation of the association between the trace element and available bioligands: proteins and compounds with relatively low molecular weight (LMW). These investigations constitute a basis of metabolic and kinetic studies and are also used to explain the mobility, storage retention and toxicity of metal ions [1]. Chemical speciation of trace metals in human blood plasma has been discussed in several review articles [2,3], but the literature on speciation of metal ions in biological fluids, as a whole, is rather limited. There were two general approaches to the problem of metal speciation: the experimental one and by computer simulation. The experimental approach is focused on developing the instrumental methods for the determination of total and free metal ion concentrations, fraction bond to proteins and fraction of total metal distributed between various LMW ligands. However, since the most of the developed experimental methods considerably disturb the labile equilibria between metal ions and LMW ligands, they are not capable of accurately determining the very low levels of complex species concentrations existing in biological matrices [4]. Thus, biospeciation of trace metal ions is commonly studied by computer simulation. Two approaches are common: (a) minimization of total Gibbs-free energy of the multi-component system, subjected to the constraints of the elemental mass balance. This approach is used in geochemical and chemical engineering simulations; and (b) equilibrium calculations based on calculation of concentrations by solving the system of mass balance equations. This approach is often used in solution chemistry and requires knowledge of the identity and stability of all metal complexes presented in biological compartment (e.g. human plasma). The aim of the computer simulation of trace element distribution in human blood plasma is to calculate the relative percentages of all presented LMW complexes of metal ions and to assess their distribution under various conditions. This task is of great significance owing to the important role of these complexes in many physiological processes taking place in blood plasma. According to May *et al.* [5], trace metals in blood plasma may be classified into four fractions: (a) non-exchangeable *i.e.* tightly bound to the metaloproteins; (b) exchangeable, loosely bound by proteins; (c) complexed by LMW ligands; (d) free (hydrated) metal ions. To calculate the concentration of LMW complexes the binding constants of metal–protein complexes are required, but the relative percentage distribution of trace metal ions amongst the LMW ligands is not controlled by protein binding and so the metal protein equilibria can be bypassed in the plasma model [6].

Metal ions enter human body mainly from food and drink but their sources may also include industrial particulate matter, medicines, *etc.* In blood plasma, they are transported through specific intestinal and/or gastric channels in the form of free (aquated ions) or complexed species with various types of endogenous or exogenous ligands [7].

Copper is an essential element which plays a critical role in human metabolism. Although, copper exists in both of its common oxidation states Cu^2+^ and Cu^+^, so far reported models of copper speciation in human blood plasma have mainly focused on Cu^2+^ speciation. In blood plasma, 65% of the copper is irreversibly bond to ceruloplasmin (non-exchangeable), ~12% to transcuprein, ~12% is loosely bond to albumin, and a small amount is distributed amongst LMW complexes [8]. It is well established that mixed ligand ternary complexes with histidine are predominant. It was assumed by Ho *at al.* [9] and May *et al.* [5] that the total Cu^2+^ concentration in plasma is 1.6× 10^−11^ mol/L and free concentration is 5.1 × 10^−16^ mol/L.

Nickel is an essential constituent of methyl-CoM reductase, CO dehydrogenase and hydrogenase in some strains of bacteria. Its physiological role in human organism is not yet fully elucidated. It is capable of activating or inhibiting a number of enzymes, altering cell membrane properties and influencing various redox processes. The main carrier proteins of nickel in serum are albumin and nickeloplasmin, but it can also be bound to α-2-macroglobulin. Plasma concentration is 10^−7^–10^−9^ mol/L and free concentration in plasma is ~1.0 × 10^−11^ mol/L. Of all LMW ligands, nickel is most strongly bound to histidine [10].

Zinc is an essential trace element whose total concentration in blood plasma is 1.6 × 10^−5^ mol/L and free concentration 10^−9^ mol/L. Zn^2+^ is rigidly bound to α-2-macroglobulin and as exchangeable fraction to albumin and serum transferrin. Its main LMW complex is with cysteine and ternary complexes with cysteine and histidine [11].

Fluoroquinolones (FQ) are synthetic antibacterial agents which could act as the competitive LMW ligands for trace metal ions in blood plasma [12,13,14]. They may remove metal ions from their protein complexes and compete with other naturally occurred ligands in blood plasma for metal binding. Its relative ability to compete for metal ions in blood plasma can be assessed in terms of the plasma mobilizing index (PMI) proposed by May and Williams [15]. The PMI may be used to determine fluoroquinolone effectiveness at mobilizing Cu^2+^, Ni^2+^ or Zn^2+^ in blood plasma. PMI is defined as:

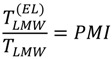
(1)
where 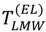 is the total concentration of the LMW metal ion fraction in the presence of exogenously administered chelating agents and *T_LMW_* is the total concentration of the LMW metal ion fraction in normal plasma. The PMI may serve to compare mobilizing power of various ligands toward particular metal ion. It permits assessment of the capacity of exogenous ligand to increase the amount of the LMW metal ion fraction at the expense of the protein bound metal. The percentage distribution of metal ions in the LMW fraction is computed assuming that the metal-protein complex is buffer free metal ion concentrations in plasma.

Based on our previously described blood plasma model [16], the aim of the present work was to establish the ability of some members of fluoroquinolone antibacterial agents to mobilize trace metal ions from blood plasma. The obtained data are essential for elucidating the mechanism of action of fluoroquinolones and the influence of metal ions on this mechanism. In addition the obtained results may be used to assess the safety of FQs toward mobilization of metal ions and consequent change of their metabolic pathways.

In this work we studied the effect of a number of FQs (ciprofloxacin-Cipro, ofloxacin-Oflo, levofloxacin-Levo and moxifloxacin-Moxi) on the biodistribution of Cu^2+^, Ni^2+^ and Zn^2+^ ions using the equilibrium calculations method. The goal of this investigation was to evaluate the capacity of some fluoroquinolone antibacterial agents to influence the LMW metal ions fraction in plasma and rationalize their ability to compete for metal ions with endogenous plasma ligands.

## 2. Results and Discussion

### 2.1. Potentiometric and Spectrophotometric Measurements

**Figure 1:**
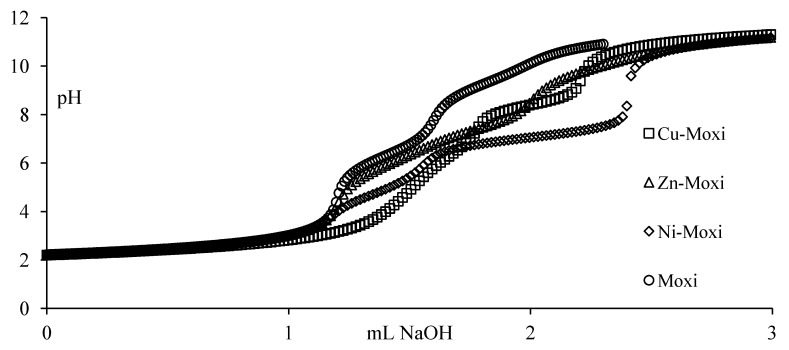
Titration curves for moxifloxacin and moxifloxacin + metal cations with NaOH at 310 K and I = 0.15 M (NaCl). Concentration of metal cations: 1.0 mmol/L, concentration of moxifloxacin: 2.0 mmol/L.

The protonation equilibria of FQ anions were studied before complexation measurements using both potentiometry and spectrophotometry, under the same conditions as used in the complexation study. The complex formation constants between metal cations (Cu^2+^, Ni^2+^ and Zn^2+^) and quinolones were investigated by emf titrations in 0.15 M (NaCl) solution at T = 310 K. Representative titration curves obtained for solutions containing moxifloxacin and metal cations are illustrated in Figure 1.

The pH titration of moxifloxacin in diprotonated form shows that the two protons dissociate in individual steps at *a* = 1–2 and *a* = 2–3 corresponding to the dissociation of the carboxyl and amine groups, respectively. The titration parameter *a* represents the number of moles of strong base added per mole of quinolone and was calculated through the formula:

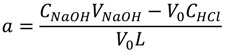
(2)
where *V_0_* and *L* are the initial volume and concentration of fluoroquinolone in the titrated solution. Below *a* = 1 strong acid (HCl) is titrated. It can be seen that moxifloxacin is present in solution in the whole titration range. In the presence of metal cations the titration curves are shifted due to the formation of metal complexes. These curves showed that complex formation begins from a low pH value and mainly occurs in the deprotonation region of the carboxyl group of moxifloxacin. The titration curves patterns of other FQs were similar to those of moxifloxacin. Precipitation was observed at pH values between pH 7–8. When precipitates formed the titrations were stopped and the corresponding points were excluded from calculations.

**Figure 2:**
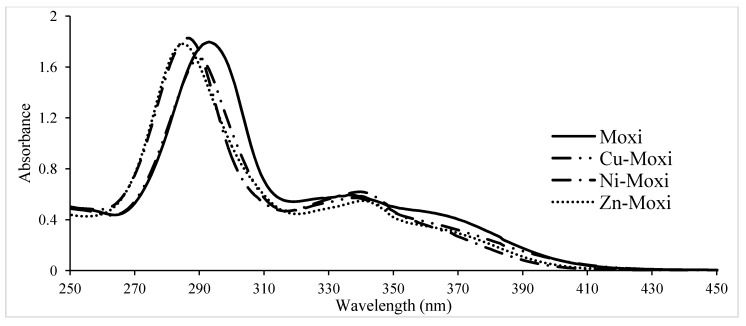
UV-Vis spectra of Cu^2+^-moxifloxacin solutions against free moxifloxacin as a blank. (C_Cu_ = 2.5 × 10^−5^ mol/L, C_Moxi_ =5 × 10^−5^ mol/L, I = 0.15 mol/L NaCl, T = 310 K).

To complement potentiometric measurements, spectrophotometric data were also obtained for metal-quinolone systems. The UV spectra in the presence of the metal cations of all FQs studied were always compared with the corresponding UV spectra of the free FQs. Spectral measurements of metal (II) + FQ solutions show evidence of an intensive higher energy band situated between 285 and 295 and lower energy broad band between 320 and 380 nm for all M^2+^ + FQ systems (Figure 2). Upon increasing the pH from 3 to 9 the higher energy band shows only small changes in position and maximum intensity (hypsochromic shift). The lower energy band exhibits however, significant changes in a shape, position and intensity (bathochromic shift). Wavelengths of maximum absorptions of free quinolones and metal-quinolones solutions are given in Table 1. In comparison with the free FQ spectra, the higher energy band may be attributed to fluoroquinolone nucleus while the lower energy band may be attributed to metal carbonyl and metal carboxyl bonds overlapping absorption. The presence of isosbestic points can be attributed not only to equilibria between protonation forms of the ligand but also proved that the different complexes were formed in solution. In the presence of metal ion, in comparison with the spectrum of fluoroquinolone alone, all bands are shifted toward higher wavelengths (Table 1).

**Table 1:** Maximum UV absorption of fluoroquinolones and their complexes at pH = 7.4.

	λ_1_^max^ (nm)	λ_2_^max^ (nm)
Moxifloxacin	289	340
Cu-Moxi	292	336
Ni-Moxi	288	340
Levofloxacin	288	332
Cu-Levo	292	326
Ni-Levo	288	330

Stability constants were calculated with Hyperquad2006 suite of programs [17]. To find the model that gives the best fit to the experimental data, various complexes and combinations thereof were included in calculations. During the calculations, the analytical parameters (total metal, ligand and proton concentration) were held constant. The pure hydrolytic complexes and protonated fluoroquinolone species were not refined during the calculations. First, the potentiometric and spectrophotometric data were separately calculated until the best set of complexes was found. For spectral data the program pHab was utilized. Then both kinds of data were treated together in Hypequad2006. The calculated protonation constants are presented in Table 2. The calculated overall stability constants of complexes are given in Table 3.

They are in good agreement with so far published data. From Table 3 it can be seen that in all systems M(FQH)^2+^ and M(FQ)_2_^0^ complexes were found. Overall stability constants of fluoroquinolones with these divalent metal ions follow the Irving-Williams series as expected (Cu ≥ Ni > Zn).

**Table 2:** Calculated protonation constants (logβ ± SD) for fluoroquinolones compared with literature values.

FQ	Methods	logβ_1_^H^	logβ_2_^H^	Ionic Medium	T °C	Reference
Cipro	UV/POT	8.81	14.71	0.15M NaCl	37	this work
	POT	8.95	15.10	0.1M NaCl	25	[18]
	UV	8.89	14.79	0.05M NaAc	25	[19]
	UV	8.73	14.91	0.2 M NaCl	25	[20]
	UV	8.84	15.17	/	/	[21]
	POT	7.41	13.55	0.1M NaNO_3_	/	[22]
	POT	8.62	14.71	0	25	[23]
Oflo	UV/POT	8.27	14.31	0.15M NaCl	37	this work
	POT	8.60	14.70	0.1M NaCl	25	[18]
	UV	7.18	/	/	/	[24]
	POT	8.28	14.38	/	/	[24]
	UV	8.28	14.25	0.05NaAc	25	[19]
	POT	8.11	14.16	0	25	[23]
	POT	7.88	/	0.1M NaNO_3_	/	[23]
Moxi	UV/POT	9.27	15.58	0.15M NaCl	37	this work
	POT	9.53	15.76	0.1 M NaCl	/	[18]
	POT	9.29	15.54	0.15 M NaCl	/	[25]
	UV	8.72	14.88	/	25	[26]
	POT	9.53	15.76	0.1M NaCl	25	[27]
	UV/POT	9.32	15.67	0.1 M LiCl	25	[28]
	UV	9.3	15.57	0.1 M LiCl	25	[29]
	POT	9.34	15.67	0.1 M LiCl	25	[29]
Levo	UV/POT	7.90	13.68	0.15M NaCl	37	this work
	POT	8.15	14.17	0.1M NaCl	25	[18]
	POT	8.15	14.17	0.1MNaCl	25	[30]

**Table 3:** Calculated overall stability constants (logβ_pqr_ ± SD) for complexes present in M^2+^-FQ (M_p_FQ_q_H_r_) systems at physiological conditions (T = 37 °C and I = 0.15 M NaCl).

	Cu-FQ			
**p q r**	**Ciprofloxacin**	**Levofloxacin**	**Ofloxacin**	**Moxifloxacin**
1 1 1	14.89 ± 0.22	13.74 ± 0.04	14.21 ± 0.03	15.77 ± 0.03
1 2 −1	6.13 ± 0.21	1.66 ± 0.06	1.88 ± 0.04	6.71 ± 0.04
1 2 0	16.06 ± 0.11	11.08 ± 0.03	11.31 ± 0.02	12.92 ± 0.02
1 2 1	22.6 ± 0.22	19.18 ± 0.04	19.42 ± 0.03	23.51 ± 0.03
1 2 2	29.06 ± 0.15	26.25 ± 0.03	26.56 ± 0.03	29.89 ± 0.03
statistics	s = 1.87	s = 0.54	s = 0.78	s = 1.23
	χ^2^ = 12.46	χ^2^ = 5.46	χ^2^ = 3.54	χ^2^ = 5.49
**Ni-FQ**				
**p q r**	**Ciprofloxacin**	**Levofloxacin**	**Ofloxacin**	**Moxifloxacin**
1 1 1	14.92 ± 0.22	13.76 ± 0.04	16.61 ± 0.03	12.72 ± 0.03
1 2 −1	6.32 ± 0.21	8.48 ± 0.06	8.84 ± 0.04	4.62 ± 0.04
1 2 0	16.95 ± 0.11	16.3 ± 0.03	16.62± 0.02	13.87 ± 0.02
1 2 1	22.34 ± 0.22	23.89 ± 0.04	24.23 ± 0.03	19.52 ± 0.03
1 2 2	28.98 ± 0.15	29.18 ± 0.03	29.58 ± 0.03	27.54 ± 0.03
statistics	s = 1.47	s = 0.93	s = 1.28	s = 1.03
	χ^2^ = 11.21	χ^2^ = 4.43	χ^2^ = 2.57	χ^2^ = 7.52
**Zn-FQ**				
**p q r**	**Ciprofloxacin**	**Levofloxacin**	**Ofloxacin**	**Moxifloxacin**
1 1 1	12.74 ± 0.22	14.2 ± 0.04	12.9 ± 0.03	12.47 ± 0.03
1 2 0	10.49 ± 0.11	9.68 ± 0.03	9.08 ± 0.02	10.56 ± 0.02
1 2 1	17.82 ± 0.22	18.04 ± 0.04	17.21 ± 0.03	17.95 ± 0.03
1 2 2	24.84 ± 0.15	24.26 ± 0.03	23.87 ± 0.03	24.91 ± 0.03
statistics	s = 0.97	s = 1.55	s = 1.18	s = 1.83
	χ^2^ = 12.46	χ^2^ = 6.41	χ^2^ = 6.34	χ^2^ =3.92

### 2.2. Reliability of the Data

In Hyperquad2006 program the quality of the fit was evaluated using the set of statistical parameters, *U*, *χ^2^*, s and *σ* [17]. The calculated set of statistics generally indicate good overall fit of the experimental data. In addition the residuals in potentials were analyzed for normal probability distribution autocorrelations and systematic trends. Inspections of the residuals in potentials for the metal-FQ model that fits best experimental data shows a linear normal probability plot and a small autocorrelation length. Examination of the residuals scatter reveals that no significant systematic trends exist, so the accepted set of complexes and their stability constants gives a plausible explanation of the experimental data. Similar results were obtained with analysis of absorbance residuals.

Existing literature data on stability constants of the studied metals with some fluoroquinolones in different media and temperature ranges are given in Table 4. Comparing our and literature models reasonably good agreement is evidenced. Complexes (111), (122), (121), (120) and (12−1) were found in all studied systems except in Zn-FQ systems where (12−1) was not found. The complex (1,1,0) has not been found in our calculations and the (1,3,2) complex of nickel with ciprofloxacin was not found either. The stability constants of 1:1 and 1:2 complexes (metal to ligand) are moderately high. Owing to the low total concentrations of metals and ligands no polynuclear complexes were found. However, protonated and hydrolytic complexes are common for all systems. The proton probably resides on secondary or tertiary nitrogen in C-7 substituent, while hydrolytic species originate from the protolysis of coordinated water. The complex [M(FQ)_2_H_2_] possesses logβ in the range ca. 26‒29 for Cu and Ni ions while with Zn ion logβ is considerably smaller.

**Table 4:** Literature overview of stability constants of the complexes M_p_(FQ)_q_H_r_ calculated from potentiometric measurements data of M(II)-FQ systems (I = ionic strength of the medium, mol/L).

Metal Ion	FQ	p q r	logβ_pqr_	I	T (°C)	Reference
Cu	Oflo	1 1 1	12.34	0.1 NaCl	25	* Gameiro *et al.* [31]
		1 2 2	23.4			
		1 1 1	14.21	0.1 NaCl	22	Urbaniak *et al.* [32]
		1 2 2	26.56			
		1 2 1	19.42			
		1 2 0	11.31			
		1 2 −1	1.88			
		1 1 1	14.84		25	* Feio *et al.* [18]
		1 2 2	28.40			
	Cipro	1 1 0	6.2	0.15 NaCl	25	Wallis *et al.* [33]
		1 1 0	6.1		37	
		1 2 0	11.1		25, 37	
		1 2 −1	4.0		25	
		1 2 −1	4.5		37	
		1 1 1	14.73	0.2 KCl	25	Turel *et al.* [34]
		1 2 2	28.53			
		1 2 1	21.93			
		1 1 1	14.43	0.1 NaCl	25	* Feio *et al.* [18]
		1 1 0	6.46			
		1 2 0	11.77			
		1 1 1	14.89	0.1 NaCl	22	Urbaniak *et al.* [32]
		1 2 −1	6.13			
		1 2 0	16.06			
		1 2 1	22.60			
		1 2 2	29.06			
	Levo	1 1 1	13.74			
		1 2 −1	1.66			
		1 2 0	11.08			
		1 2 1	19.18			
		1 2 2	26.25			
		1 1 1	14.37		25	* Feio *et al.* [18]
		1 1 0	7.13			
		1 2 2	27.22			
		1 2 0	11.78			
	Moxi	1 1 1	15.54			
		1 1 0	8.21			
		1 2 2	31.30			
		1 2 0	14.17			
		1 1 1	14.21			
		1 1 0	10.20			
		1 2 2	27.73			
		1 2 0	22.17			
Ni	Oflo	1 1 1	13.42			
		1 1 0	5.91			
		1 2 2	25.60			
	Cipro	1 1 1	13.41			
		1 1 0	7.43			
		1 1 1	12.93	0.2 KCl	25	Turel *et al.* [34]
		1 2 2	25.77			
		1 2 1	18.42			
		1 3 2	29.7			
Zn		1 1 1	12.32	0.2 KCl	25	
		1 2 2	24.72			
		1 2 1	17.41			
		1 1 1	12.74	0.1 NaCl	22	Urbaniak *et al.* [32]
		1 2 0	10.49			
		1 2 1	17.82			
		1 2 2	24.84			
	Oflo	1 1 1	14.64			
		1 2 0	10.51			
		1 2 1	18.36			
		1 2 2	24.40			
	Levo	1 1 1	12.90			
		1 2 0	9.08			
		1 2 1	17.20			
		1 2 2	23.87			
	Oflo	1 1 1	12.67		25	* Feio *et al.* [18]
		1 1 0	5.23			
		1 2 2	24.48			

* Values of stability constants were recalculated taking into account M, H and L as components instead of M and HL as given in the original reference.

### 2.3. Solution Equilibria and Distribution Diagrams

**Figure 3:**
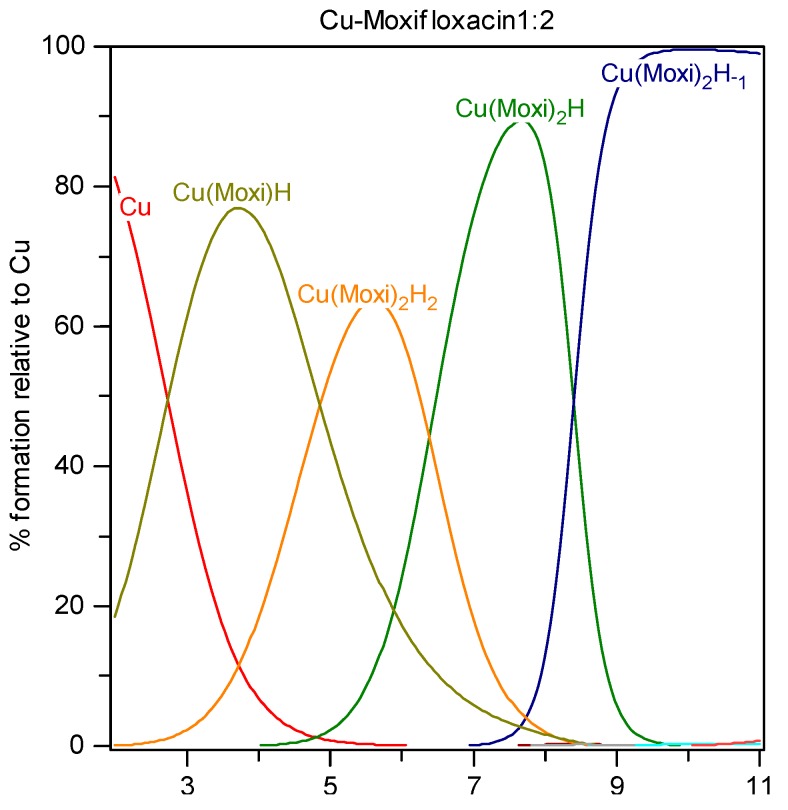
Distribution diagram of Cu-moxifloxacin species at ligand-to-metal concentration ratio 2:1 and total metal concentration 1.0 mmol/L.

**Figure 4:**
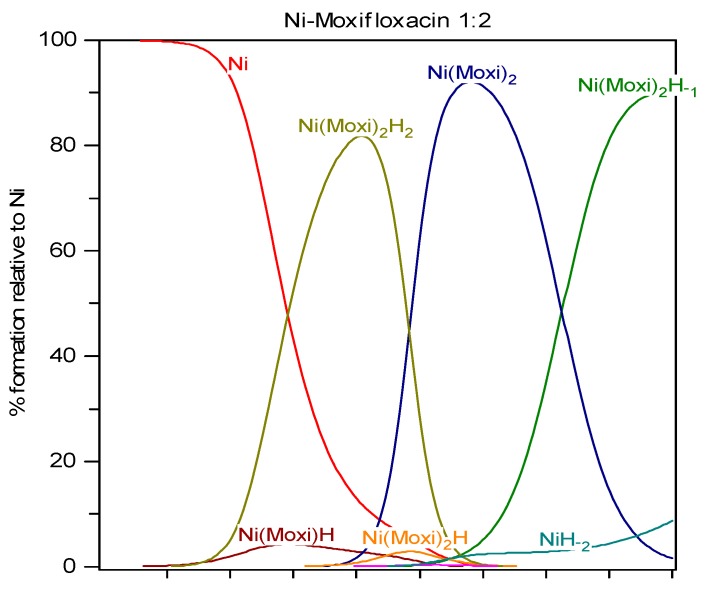
Distribution diagram of Ni-moxifloxacin species at ligand-to-metal concentration ratio 2:1 and total metal concentration 1.0 mmol/L.

**Figure 5:**
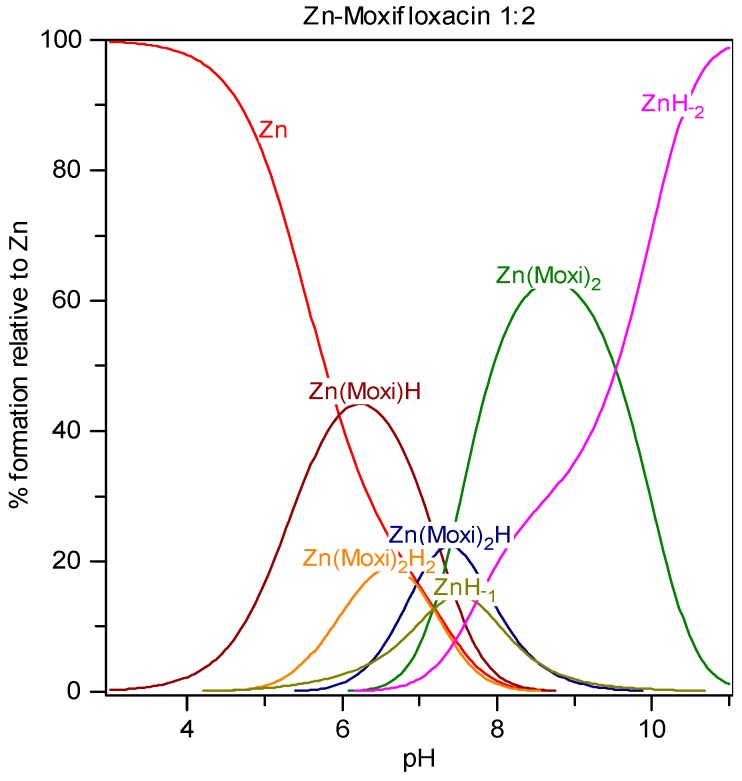
Distribution diagram of Zn-moxifloxacin species at ligand-to-metal concentration ratio 2:1 and total metal concentration 1.0 mmol/L.

The distribution of various complexes in M(II)-FQ solutions are shown in Figure 3, Figure 4 and Figure 5. In the following text the complexes are designated with an acid HMoxi as a component to make the mechanisms meaningful. HMoxi may be either neutral or a zwitterion, the latter being more probable. As can be seen from Figure 3, at lower pH values (less than 3), hydrated copper ion exists dominantly in solution. With increasing pH values over 3 the formation of Cu(HMoxi)^2+^ complex (Cu(Moxi)H on the distribution diagram) starts, probably according to the reaction:

Cu^2+^ + HMoxi^0^ ↔ [Cu(HMoxi)]^2+^ (3)

with the maximum concentration at pH = 4. This complex upon increasing pH, binds another molecule of moxifloxacin and gives [Cu(HMoxi)_2_]^2+^ complex (Cu(Moxi)_2_H_2_ on the distribution diagram) with maximum concentration at pH ~6, by the reaction:

[Cu(HMoxi)]^2+^ + HMoxi^0^ ↔ [Cu(HMoxi)_2_]^2+^ (4)

At pH values above 6.5 this complex releases protons and gives the mixed complex [Cu(HMoxi)Moxi]^+^ (Cu(Moxi)_2_H on the distribution diagram) with a maximum concentration at pH values close to physiological. Further pH increases lead to the release of another proton and formation of [Cu(Moxi)_2_(OH)] species (Cu(Moxi)_2_H_−1_ on the distribution diagram) at pH > 8. The complex [Cu(Moxi)_2_]^0^ is a minor species between pH value 7.5–8.5. The overall complex reaction follows the scheme:

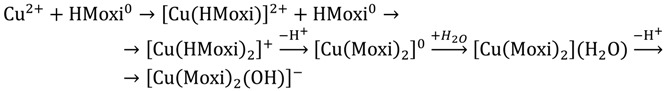
(5)


Copper(II) ion exhibits characteristic coordination numbers 4, 5 and 6. In the [Cu(HMoxi)_2_]^2+^ complex, according to X-ray data [35], moxifloxacin acts as a bidentate O,O-ligand with probable formation of a six-membered ring by the 4-keto and 3-carboxyl oxygens [36,37,38,39,40]. It may be assumed that one or two additional coordination sites in the copper coordination sphere are occupied with water molecules. One of water molecules is more strongly bound to Cu^2+^ ion and is subject to hydrolysis upon increasing the pH. The other water molecule hydrolyzes at pH > 11 and this leads to the onset of precipitation.

Figure 4 represents the distribution of moxifloxacin species in solution in the presence of nickel. Obvious dominant species are those of the ML_2_ type. [Ni(HMoxi)_2_]^2+^ complex (Ni(Moxi)_2_H_2_ on the distribution diagram) forms in the pH 4–7 range with the maximum concentration at pH = 6 and neutral complex [Ni(Moxi)_2_]^0^ in pH range 6–10 with maximum concentration at pH = 8. The complex [Ni(Moxi)_2_]^0^ is very stable in the 7.0–9.0 pH range and is probably formed in plasma under physiological conditions. The complexation behavior of moxifloxacin toward nickel is important for understanding the antibacterial action of the drug against metal-dependent microorganisms. For example, *Helicobacter pylori* (*H. pylori*) is a common human pathogen responsible for various gastric diseases. This bacterium produces urease and hydrogenase to survive in the acidic environment of the stomach. Nickel is an essential cofactor for urease and hydrogenase. *H. pylori* has to uptake sufficient nickel ions [41] for the maturation of urease, and on the other hand, to prevent the toxic effects of excessive nickel ions. Moxifloxacin was recently found to be effective against *H. pylori*. By binding the Ni^2+^ into a strong Ni-Moxi complex, moxifloxacin acts as safe and strong *Helicobacter pylori* eradicator [42].

The distribution diagram of species in the Zn^2+^-moxifloxacin system, for the concentration ratio [Moxi]/[Zn] = 2:1 is shown in Figure 5. As can be seen from Figure 5 the dominating complexes in a wide pH range are [Zn(HMoxi)]^2+^ (Zn(Moxi)H on the distribution diagram) and [Zn(Moxi)_2_]^0^, with the maximum concentrations at pH 6 and *ca.* 9, respectively. These complexes may be formed via the same reaction mechanism as in case of copper and nickel ions. In the pH range 5.5–8.5 the complexes [Zn(HMoxi)_2_]^2+^ (Zn(Moxi)_2_H_2_ on the distribution diagram) and [Zn(HMoxi)(Moxi)]^+^ (Zn(Moxi)_2_H on the distribution diagram) occur to a lesser extent. Hydrolytic species of Zn^2+^ disturb the complexation considerably. Micro-colloidal precipitated ZnH_-2_ is dominant at pH > 9. Similar species distribution results were obtained for the other M-FQ systems.

### 2.4. Fluorescence Measurements

Spectrofluorimetric titrations were carried out by addition of known quantities of metal ion (concentration range (0–1) × 10^−3^ mol/L) to 2 × 10^−5^ mol/L buffered solutions of the ligand.

**Figure 6:**
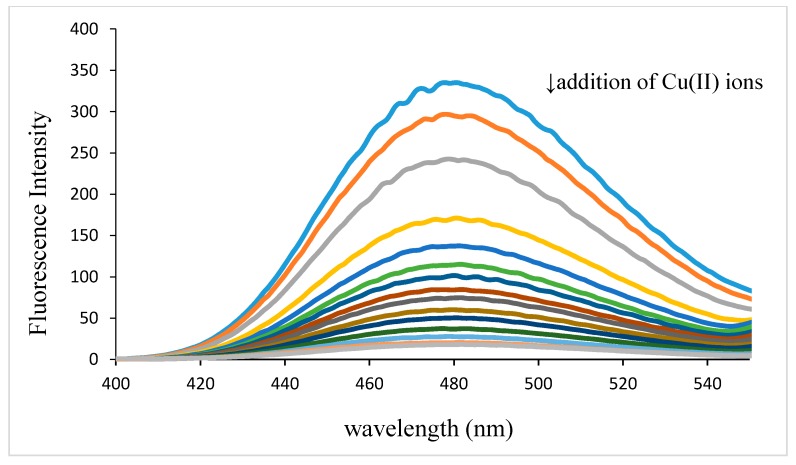
Fluorescence emission spectrum of levofloxacin (2 × 10^−5^ mol/L, λ_ex_ = 292 nm) in aqueous solution at pH 7.4 in the presence of Cu(II) ions (concentration range of Cu ion: (0–1) × 10^−3^ mol/L).

The wavelenght of excitation was 292 nm (for levofloxacin). Fluorescence spectra of levofloxacin in the absence and the presence of copper ion are presented in Figure 6. From the figure a decrease of fluorescence intensity upon addition of copper ion can be seen. The maximum emission of levofloxacin occurs at 482 nm and it shifts hypsochromically slightly upon addition of copper.

**Figure 7:**
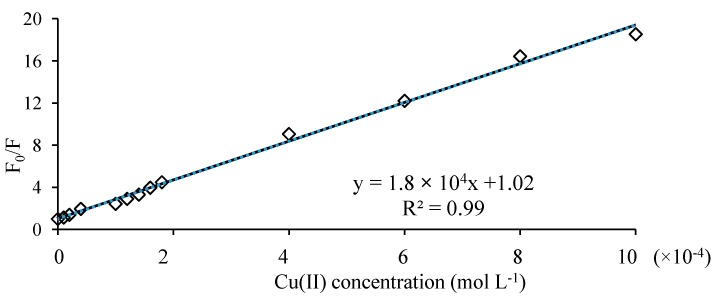
Stern-Volmer plot for the quenching of levofloxacin by copper ion.

To examine the fluorescence quenching mechanism a Stern-Volmer plot was used and presented in Figure 7.

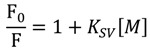
(6)
where F_0_ and F are the fluorescence intensity in the absence and the presence of the quencher, respectively and [M] is the concentration of the metal ion [43]. From the slope of the Stern-Volmer plot, the Stern-Volmer constant can be determined. The obtained value was K_SV_ = 1.82 × 10^4^ or logK_SV_ = 4.26. Fluorescence quenching typically occurs through either static or dynamic quenching mechanisms. The static quenching results in a linear relationship between (F_0_/F) and concentration of metal ion. This result suggests that levofloxacin fluorescence quenching in the precense of copper ion was probable static quenching as a result of the formation of a non-fluorescent complex between the fluorophore and the quencher.

*Stability constant determination*. The complexation of a metal ion Cu^2+^ by a ligand levofloxacin in solution can be represented by the cumulative equilibria:

M + L ↔ ML
(7)

M + 2L ↔ ML_2_ (8)

with the association constant K:

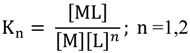
(9)
expressing the degree of stability of the complex in the given solvent and temperature conditions. The stability constants of the formed complexes can be calculated from the double reciprocal plot:

For 1:1 complex:

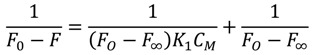
(10)


**Figure 8:**
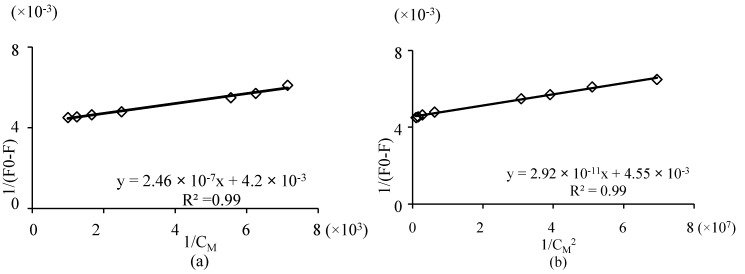
Double reciprocal plot obtained from 1/(F − F_0_) plotted against: (**a**) 1/C_M_ and (**b**) 1/C_M_^2^.

And for 1:2 complex:

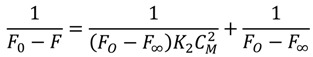
(11)
where F_0_ and F are the fluorescence intensity of ligand in the absence of metal ion and fluorescence intensity of solution at each metal concentration, respectively [44,45]. F_∞_ is the fluorescence intensity when fluorescence of the solution remains constant with further addition of metal ion. K_1_ and K_2_ are the stability constants of the formed complexes. When 1/(F_0_ − F) is plotted against C_M_^−1^ or C_M_^−2^ (Figure 8) the stability constant is calculated by the ratio intercept/slope. The formation stability constant for these complexes calculated to be logK_1_ = 4.23 and logK_2_ = 8.19 or logβ_1_ = 4.23 and logβ_2_ = 12.42. These values are very close to those calculated by potentiometric and spectrophotometric measurements.

#### Consideration of the Inner Filter Effect

UV absorbance spectra demonstrate that FQs and their metal complexes strongly absorb at 292 nm, the excitation wavelenght of FQs. The attenuation of the excitation beam due to such absorption results in a fluorescence intensity decrease know as the inner filter effect [46]. Since absorbtion is high correction was aplied to all our fluorescence data. The corection factor CF was calculated according to Parker and Barnes equation [47]:

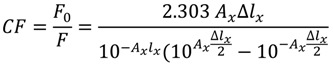
(12)
where F_0_ is the corrected fluorescence, F is the observed fluorescence and A_x_ is the optical density of the solution at excitation wavelength; *l*_x_ and ∆*l*_x_ are geometrical parameters of the fluorimetric cell. The *l*_x_ was calculated according to formula *l*_x_ = (1 − ∆*l*_x_)/2. ∆*l*_x_ was set as 0.250 cm corresponding to 10 nm bandwidth [48]. The calculated correction factor was in the range 1.50–1.73.

### 2.5. H^1^-NMR Measurements

**Figure 9:**
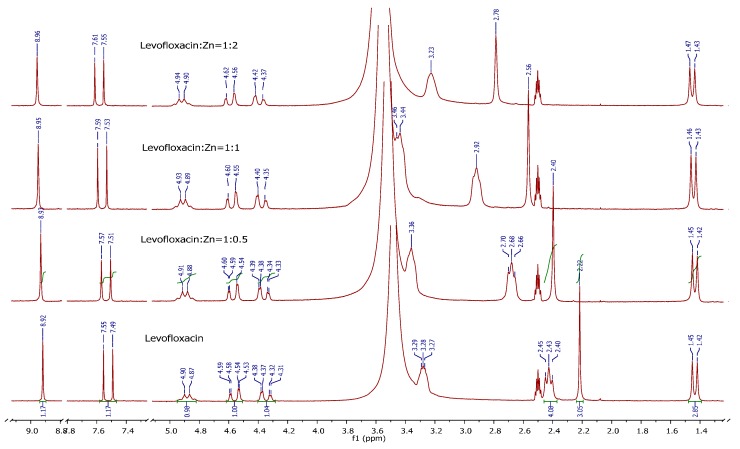
^1^H-NMR spectra of Zn(II)-levofloxacin complexes at various mole ratios of the ligand and zinc salt in DMSO-d_6_ solution recorded at 200 MHz.

To extend the speciation study some NMR spectra of the studied systems were recorded. The NMR experimental conditions were so chosen to make speciation unambiguous. Proton NMR spectra of levofloxacin and Zn^2+^ + levofloxacin at different molar ratios are shown in Figure 9.

**Figure 10:**
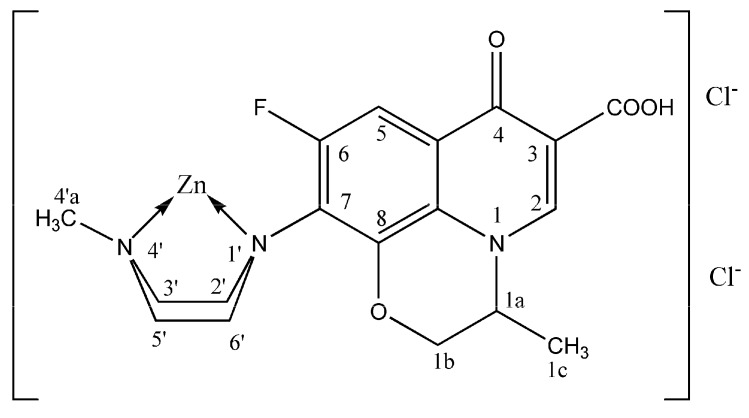
Proposed structure for Zn(II)-levofloxacin complex.

The signals for aryl protons (doublet at 7.52 (C-5) and singlet at 8.92 ppm (C-2)), oxazine protons (4.31–4.90 ppm) and methyl group protons attached to oxazine ring (doublet at 1.43 ppm) are practically unchanged after the addition of different amounts of zinc(II)-chloride. This indicates that they are situated far from the binding site. However, two groups of the piperazine signals as well as the methyl protons of the piperazine nitrogen appeared at significantly higher δ-values in all ^1^H-NMR spectra of the zinc(II)-complexes. This strong downfield shift for the piperazine methyl group (0.56 ppm), 3',5'-piperazine protons (0.79 ppm) and 2',6'-piperazine protons (more than 0.3 ppm, overlapped with the water peak) indicates that the piperazine nitrogen atoms are coordinated to the zinc (Figure 9). A tentative structure of the complex may be represented as in Figure 10.

Addition of Zn^2+^ into a D_2_O solution of levofloxacin at pH = 6.0 (mole ratio: Zn to Levo= 1:1 and 2:1) produces C-2 proton shift toward lower field (Table 5). Most other proton signals are also shifted downfield in comparison with free ligand protons. It may be concluded that zinc is coordinated to the carboxylate and 4-carbonyl oxygen in D_2_O as reported in many papers [36,37,38,39,40]. Thus, the binding sites of Zn^2+^ ion in D_2_O are different from those in DMSO-*d*_6_. The difference may be attributed to the different polarity of these molecules.

**Table 5:** ^1^H-NMR chemical shifts of Levo and Levo + Zn^2+^ (concentration ratio 1:1 at pH = 6.0).

Levo-proton	δ _(free)_	δ _(complex)_	∆δ ^a^
H-2	8.34	8.51	0.17
H-5	7.24	7.25	0.01
H-1a, 1b	4.36–4.61	4.38–4.64	0.01
H-3', 5'	3.50	3.61	0.11
H-2', 6'	3.38	3.46	0.08
H-4'a	2.95	2.99	0.04
H-1c	1.44	1.43	0.01

^a^ ∆δ = δ_(complex)_ − δ_(free)__._

### 2.6. ESI-MS Measurements

**Figure 11:**
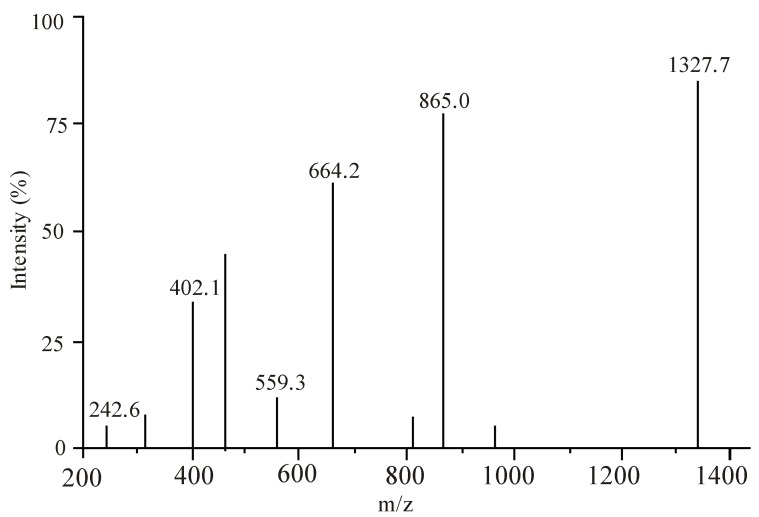
ESI-MS spectrum of Cu-moxifloxacin solution at pH = 4.5.

To further confirm the speciation derived from potentiometric and spectrophotometric measurements ESI-MS measurements were made on fluoroquinolone-metal ion solutions. The experimental conditions were adjusted in such a way that the fragmentation is minimal. Several time repeated measurements resulted in nearly the same spectra, indicating that the obtained signals belong to species formed in solutions rather than in gas plasma. The most intensive signals can be attributed to the 1:1 and 1:2 (metal to ligand) complexes. Signals arising from fragmentation can be explained either by CO_2_ or H_2_O loss. Loss of HF was not observed. The ESI-MS data of M-FQ solutions with FQ to metal concentration ratio 2:1 at pH 4.5 show evidence to formation of the complex M(FQ) and M(FQ)_2_ as shown in Table 6, Table 7 and Table 8, and in Figure 11.

**Table 6:** Experimental and theoretical *m/z* values of ESI-MS spectra in Copper-fluoroquinolone solution at pH = 4.5; ((*m/z*)_e_ and (*m/z*)_t_ denote experimentally determined and calculated value, respectively).

			Complex			
Fluoroquinolone	[ML]^+^		[ML_2_]^+^		[M_2_L_3_]^+^	
	(*m/z*)_e_	(*m/z*) _t_	(*m/z*)_e_	(*m/z*) _t_	(*m/z*)_e_	(*m/z*) _t_
Moxifloxacin	464.3	463.9	865.0	864.8	1327.7	1327.3
Ofloxacin	425.1	424.9	784.2	784.2	-	-
Levofloxacin	425.0	424.9	-	-	-	-
Ciprofloxacin	-	-	724.1	724.2	-	-

**Table 7:** Experimental and theoretical *m/z* values of ESI-MS spectra in Zinc-fluoroquinolone solution at pH = 4.5.

	Complex			
Fluoroquinolone	[ML]^+^		[ML_2_]^+^	
	(*m/z*)_e_	(*m/z*)_t_	(*m/z*)_e_	(*m/z*) _t_
Moxifloxacin	466.1	465.7	-	-
Ofloxacin	425.2	424.9	-	-
Levofloxacin	-	-	786.4	786.0
Ciprofloxacin	-	-	726.0	725.9

**Table 8:** Experimental and theoretical *m/z* values of ESI-MS spectra in Nickel-fluoroquinolone solution at pH = 4.5.

	Complex			
Fluoroquinolone	[ML]^+^		[ML_2_]^+^	
	(*m/z*)_e_	(*m/z*)_t_	(*m/z*)_e_	(*m/z*)_t_
Moxifloxacin	459.2	459.0	859.6	859.9
Ofloxacin	419.5	419.2	-	-
Levofloxacin	-	-	778.9	779.3
Ciprofloxacin	389.6	389.1	719.5	719.4

### 2.7. Influence of Fluoroquinolones on Bio-Speciation of Copper, Nickel and Zinc Ions in Human Blood Plasma

The LMW bio-distributions of Cu(II), Zn(II) and Ni(II) ions in the presence of fluoroquinolone in blood plasma were computed using the HySS2009 program. The total concentrations of physiological plasma ligands and metals were as described in our previous paper [16]. Average maximum concentration of quinolones in human plasma is *ca.* 2 × 10^−5^ mol/L.

**Table 9:** Calculated bio-distribution of dominant Cu(II) species (%) in human blood plasma in the presence of fluoroquinolones.

Cu-LMW Species	Concentration of Fluoroquinolone (mol/L)			
	0	1 × 10^−5^	1 × 10^−4^	1 × 10^−3^
	Moxifloxacin			
CuCisHis	25.0	23.9	23.9	22.7
CuHCisHis	15.8	15.1	15.1	14.3
CuHis_2_	10.7	10.2	10.2	9.7
CuHisSer	6.8	6.5	6.5	6.1
CuHisThr	6.0	5.8	5.8	5.5
CuHHisLys	4.5	4.3	4.3	4.1
	**Ciprofloxacin**			
CuCisHis	25.0	25.0	24.9	20.8
CuHCisHis	15.8	15.7	15.7	13.2
CuHis_2_	10.7	10.7	10.7	8.9
CuHisSer	6.8	6.7	6.7	5.6
CuHisThr	6.0	6.0	6.3	5.0
CuHHisLys	4.5	4.5	4.5	3.7
	**Ofloxacin**			
CuCisHis	25.0	25.0	25.0	25.0
CuHCisHis	15.8	15.8	15.8	15.8
CuHis_2_	10.7	10.7	10.7	10.7
CuHisSer	6.8	6.8	6.8	6.7
CuHisThr	6.0	6.0	6.0	6.0
CuHHisLys	4.5	4.5	4.5	4.5
CuCisHis	25.0	25.0	25.0	25.0
CuHCisHis	15.8	15.8	15.8	15.8
	**Levofloxacin**			
CuHis_2_	10.7	10.7	10.7	10.7
CuHisSer	6.8	6.8	6.8	6.7
CuHisThr	6.0	6.0	6.0	6.0
CuHHisLys	4.5	4.5	4.5	4.4

#### 2.7.1. Copper in Human Blood Plasma Model

**Figure 12:**
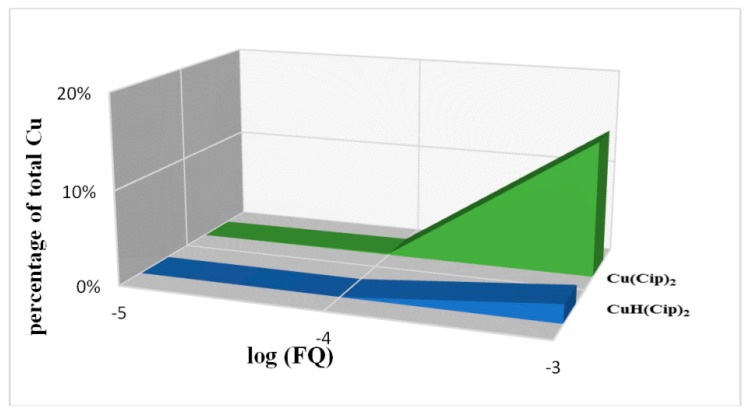
Dominant Cu-Cipro species in blood plasma in the presence of ciprofloxacin (Cip).

The concentration of free copper is taken as 1 × 10^−19^ mol/L. The major LMW complexes of copper ion in plasma at normal physiological conditions (no exogenous ligand addition) were computed and are shown in Table 9 (first column). At ligand concentrations of less than 1 × 10^−4^ mol/L the fraction of main copper complexes remains unchanged. In the case of ciprofloxacin, however, a small change in the fraction of dominant Cu-LMW species at ligand concentrations 1 × 10^−5^–1 × 10^−4^ mol/L is seen (Table 9). With increasing ligand concentration to 1 × 10^−3^ mol/L only moxifloxacin and ciprofloxacin influence the fraction of Cu-LMW species considerably. Thus, these fluoroquinolones appear as competitive ligands to amino acids. Upon further increasing the ligands concentration to 1 × 10^−2^ mol/L fractions of main LMW complexes continue to decrease until, in the extreme case, the complexes of Cu-FQ appear. Normal level of FQs in blood plasma after a single dose of a 500 mg tablet administration is *ca.* 10^−5^ mol/L [49]. This peak concentration lasts for about 2–3 h and then begins to fall. Considering the plasma life of FQs is long enough to establish a local equilibrium, competitive reactions between LMW complexes of copper ion and FQs may occur. Thus, a fraction of copper may be bound to the FQs. It is well known that metal–FQ complex species alter the properties of the FQ in terms of bioavailability and antimicrobial action [50]. As illustrated in Figure 12, increasing the plasma concentration of FQ increases the fraction of mobilized copper. It may provide an additional explanation of the mechanism of FQ action with respect to the role of essential metal ions.

**Figure 13:**
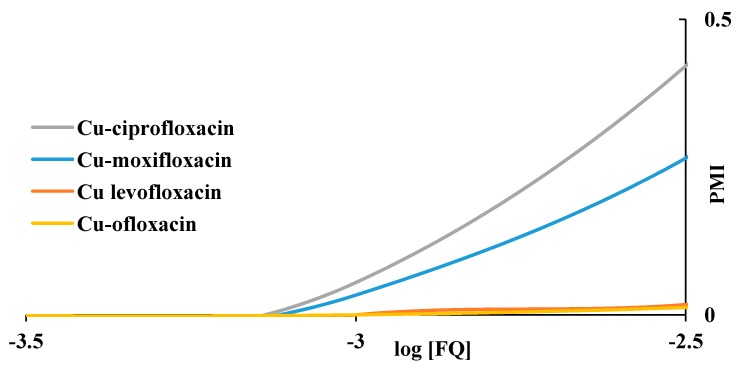
The effect of fluoroquinolone ligands on plasma mobilization of copper.

The PMI curves of copper ion with the studied fluoroquinolones are shown in Figure 13. It can be seen that ciprofloxacin has the highest mobilizing effect. The effects of ofloxacin and levofloxacin do not differ significantly.

#### 2.7.2. Nickel in Human Blood Plasma Model

Earlier works suggested that the major nickel species in blood plasma are Ni(His)_2_, Ni(Cys)(His), Ni(Cys)_2_ and Ni(His) [51]. Blood nickel levels of 1 × 10^−8^ mol/L have been reported [52]. The calculated bio-distribution of Ni-LMW species in the absence and in the presence of fluoroquinolones is presented in Table 10. As it can be seen from the data, the FQ with the strongest influence on LMW nickel complexes was levofloxacin, even at a concentration of 1 × 10^−5^ mol/L^−1^. The calculated distribution in the presence of moxifloxacin indicates its relatively lower influence on Ni-LMW species, in a wide range of concentrations, compared to Cu-LMW species. The effects of these ligands on the biodistribution of Ni-LMW species has the order levofloxacin > ciprofloxacin > ofloxacin > moxifloxacin.

**Table 10:** Calculated bio-distribution of dominant Ni (II) species (%) in human blood plasma in the presence of fluoroquinolones.

Ni-LMW Species	Concentration of Fluoroquinolone (mol/L)				
	0	1 × 10^−7^	1 × 10^−5^	1 × 10^−4^	1 × 10^−3^
	Levofloxacin				
NiHis_2_	83.2	83.2	73.0	4.9	0.0
NiCysHis	9.2	9.2	8.0	0.5	0.0
NiCys_2_	3.8	3.8	3.4	0.2	0.0
NiHis	2.4	2.4	2.1	0.1	0.0
NiHisCit	1.2	1.2	1.1	0.1	0.0
NiCys	0.1	0.1	0.1	0.0	0.0
	**Ofloxacin**				
NiHis_2_	83.2	83.2	79.7	17.0	0.2
NiCysHis	9.2	9.2	8.8	1.9	0.0
NiCys_2_	3.8	3.8	3.7	0.8	0.0
NiHis	2.4	2.4	2.3	0.5	0.0
NiHisCit	1.2	1.2	1.2	0.2	0.0
NiCys	0.1	0.1	0.1	0.0	0.0
	**Ciprofloxacin**				
NiHis_2_	83.2	83.2	77.5	9.8	0.1
NiCysHis	9.2	9.2	8.5	1.1	0.0
NiCys_2_	3.8	3.8	3.6	0.5	0.0
NiHis	2.4	2.4	2.2	0.3	0.0
NiHisCit	1.2	1.2	1.1	0.1	0.0
NiCys	0.1	0.1	0.1	0.0	0.0
	**Moxifloxacin**				
NiHis_2_	83.2	83.2	83.2	83.0	68.1
NiCysHis	9.2	9.2	9.1	9.1	7.5
NiCys_2_	3.8	3.8	3.8	3.8	3.1
NiHis	2.4	2.4	2.5	2.4	1.9
NiHisCit	1.2	1.2	1.2	1.1	1.0
NiCys	0.1	0.1	1.0	1.0	0.9

**Figure 14:**
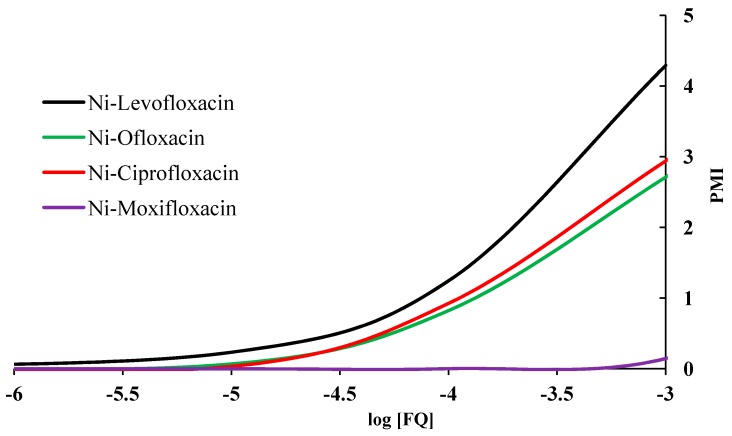
The effect of fluoroquinolone ligands on plasma mobilization of nickel.

The calculated plasma mobilizing index of nickel in the presence of FQs in shown in Figure 14. Levofloxacin shows higher PMI values than the other FQs. This surprisingly high effect may be perhaps be useful in the treatment of *H. pylori* with levofloxacin.

#### 2.7.3. Zinc in Human Blood Plasma Model

Since a 35% of total zinc is rigidly bound to the proteins the total concentration of exchangeable zinc is estimated to be about 1 × 10^−6^ mol/L [5]. Calculated distribution of LMW-Zn species is presented in Table 11. At nominal plasma levels, FQs does not exert a significant effect on LMW-Zn fractions. It seems that FQs are not effective competitors to amino acids for Zn binding.

**Table 11:** Calculated bio-distribution of dominant Zn(II) species (%) in human blood plasma in the precense of fluoroquinolones.

Zn-LMW	Concentration of Fluoroquinolone (mol/L)				
	0	1 × 10^−7^	1 × 10^−5^	1 × 10^−4^	1 × 10^−3^
species	Moxifloxacin				
ZnCysCit	39.2	39.2	39.2	39.3	38.2
ZnCys_2_	19.4	19.4	19.4	19.3	18.3
ZnCysHis	10.6	10.6	10.6	10.6	10.0
ZnHis	3.6	3.6	3.6	3.6	3.4
ZnHCys_2_	2.7	2.7	2.7	2.7	2.6
	**Levofloxacin**				
ZnCysCit	39.2	39.2	39.3	39.8	28.2
ZnCys_2_	19.4	19.4	19.3	19.0	11.5
ZnCysHis	10.6	10.6	10.6	10.4	6.0
ZnHis	3.6	3.6	3.6	3.5	1.9
ZnHCys_2_	2.7	2.7	2.7	2.7	1.6
	**Ciprofloxacin**				
ZnCysCit	39.2	39.2	39.3	39.5	37.1
ZnCys_2_	19.4	19.4	19.4	19.2	16.1
ZnCysHis	10.6	10.6	10.6	10.5	8.7
ZnHis	3.6	3.6	3.6	3.6	2.9
ZnHCys_2_	2.7	2.7	2.7	2.7	2.3
	**Ofloxacin**				
ZnCysCit	39.2	39.2	38.8	35.6	19.7
ZnCys_2_	19.4	19.4	19.2	17.8	10.3
ZnCysHis	10.6	10.6	10.5	9.6	5.2
ZnHis	3.6	3.6	3.6	3.2	1.6
ZnHCys_2_	2.7	2.7	2.7	2.5	1.5

**Figure 15:**
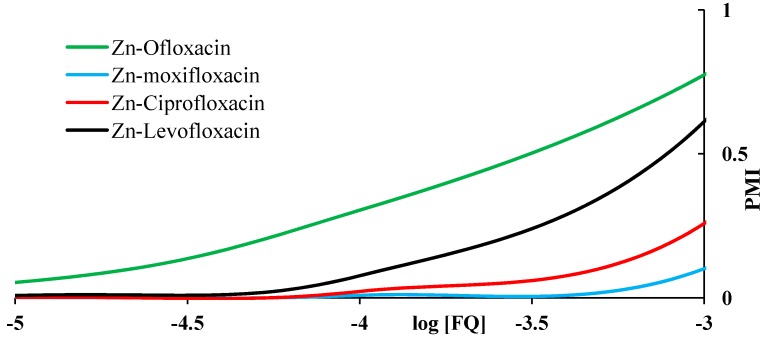
The effect of fluoroquinolone ligands on plasma mobilization of zinc.

PMI dependence on FQ’s concentration is shown in Figure 15. The FQ’s do not show much difference between themselves toward mobilizing power to Zn.

## 3. Experimental Section

### 3.1. Reagents

Moxifloxacin, (1-cyclopropyl-6-fluoro-8-methoxy-7-[(4a*S*,7a*S*)-octahydropyrolo[3,4-b]pyridine-6-yl]- 4-oxo-1,4-dihydroquinoline-3-carboxylic acid hydrochloride) and ciprofloxacin (1-cyclopropyl-6-fluoro-4-oxo-7-(piperazin-1-yl)-quinoline-3-carboxylic acid) (declared purities ˃99.9%) were obtained from Bayer Pharma AG (Berlin, Germany); ofloxacin (9-ﬂuoro-3-methyl-10-(4-methyl-1-piperazinyl)-7-oxo-2,3-dihydro-7H-pyrido-(1,2,3-de)1,4-benzoxazine-6-carboxylic acid) and levofloxacin ((*S*)-9-fluoro-2,3-dihydro-3-methyl-10-(4-methylpiperazin-1-yl)-7-oxo-7*H*-pyrido[*1*,*2*,*3*-*de*]-1,4-benzoxaz-ine-6-carboxylic acid) (≥99.9% pure) were purchased from Sigma Aldrich (St. Louis, MO, USA). Nickel(II) chloride hexahydrate (≥98%, Sigma Aldrich), copper(II) chloride dihydrate (≥99.9%, Sigma Aldrich) and zinc(II) chloride hydrate (≥98%, Sigma Aldrich) were utilized. Doubly distilled water was used for preparation of all solutions (conductivity less a 0.1 µS cm^−1^).The solutions of fluoroquinolones (5 × 10^−3^ mol/L) were prepared by direct weighing of standard substances.Metal salts were disolved in water with the addition of appropriate amount of HCl to avoid initial hydrolysis of metal ions. The concentration were determined by complexometric titration using EDTA and by electrogravimetry. The excess of HCl in the metal chloride stock solution was determined potentiometrically using Gran’s method. A sodium hydroxide solution was prepared from concentrated volumetric solutions (p.a., Merck, NJ, USA), diluted with freshly boiled doubly distilled water followed by cooling under a constant flow of purified nitrogen. The alkali concentration was checked by titration against potassium hydrogen phthalate. Hydrochloric acid solution was made from HCl “Suprapure” (Merck) and standardized against tris(hydroxymethyl) aminomethane. A sodium chloride solution was prepared from NaCl, (p.a., Merck), by dissolving the re-crystallized salt in twice-deionized water. The concentration of this solution was determined by evaporation of a known volume of solution to dryness at 573 K and weighing the residue. Nitrogen gas, used for stirring solutions and providing an inert atmosphere during the titrations, was purified by passing it through 10% NaOH then 10% H_2_SO_4_, alkaline solution of pyrogallol, 0.1 mol/Lsolution of KCl and finally distilled water.

### 3.2. Instruments

Potentiometric measurements were made on a Tacussel Isis 20000 pH meter (Courthezon, Vaucluse, France, precision ± 0.1 mV or ± 0.001 pH units) equipped with a Radiometer combined electrode. A Metrohm Dosimat model 665 automatic burette with anti-diffusion tip (Herisau, Switzerland), was used for delivery of the titrant.

UV spectral measurements were performed on a double beam UV-Vis spectrophotometer model Lambda 35 (PerkinElmer, Waltham, MA, USA). Operational parameters were: scan speed, 2 nm/s, slit width, 0.3 nm, photometric sensitivity, 0.2 abs. units. Matching pair of 1 cm quartz cuvettes was used for measuring the spectra.

Fluorescence spectra were collected on Shimadzu RF-1501 spectrofluorimeter (Kyoto, KYT, Japan) with a 150 W xenon lamp and 1.0 × 1.0 cm quartz cells. The slit width was set to 10 nm on both the excitation and emission monochromators. To ensure reproducible experimental conditions instrument performance was checked daily using quinine sulphate solution (4 g/L quinine in 0.5 mol/LH_2_SO_4_).

ESI MS spectra were collected on an LCQ Fleet 3D Ion Trap Mass Spectrometer (Thermo Fisher Scientific, Waltham, MA, USA). ^1^H-NMR spectra were recorded on Varian Gemini 200 spectrometer (Palo Alto, CA, USA).

### 3.3. Procedure

#### 3.3.1. Potentiometric Titrations

Potentiometric titrations were carried out in a double-walled glass vessel, thermostatted at 310 K. The ionic strength of all test solutions was adjusted to 0.15 mol/Lwith sodium chloride. All measurements were performed under a nitrogen atmosphere. The titration protocol was chosen in such a way that the protonation, hydrolysis and complexation reactions would proceed in the conditions as close to true equilibrium as possible. Usually stable potential readings were obtained in 3–5 min after addition of the titrant. If equilibrium could not be established within specified time interval the point was discarded. The electrode parameters, *E_0_*, *Q* and *E_j_* from Nernst equation: *E = E_0_ + Q logh + E_j_* were determined by strong acid-strong base titration to check the system suitability. During the titrations of the test solutions the *E_0_* and *E_j_* were determined using the data in the acidic region where no hydrolysis or complexation takes place (assuming that *h* is equal to the analytical concentration of proton), by plotting *E–Q log h* against *h* and extrapolating the straight line so obtained to *h = 0*. The free proton concentration was then calculated through the equation: *logh = (E – E_0_ – E_j_)/Q* which was applied to the whole titration curve. All titrations were carried in duplicate. The agreement between duplicate titration was better than 1%. The samples of FQ + metal ion solutions were titrated with sodium hydroxide and all titration were performed in the pH range from 2 to 11 with constant ionic strength (I = 0.15 mol/LNaCl) and under purified nitrogen atmosphere at 310 K. Molar ratios between metal ions and fluoroquinolones ranged from 1:1 to 1:2 for all M-FQ systems.

#### 3.3.2. Spectrophotometric Measurements

Spectra of solutions of FQ alone (5 × 10^−5^ mol/L) and FQ+metal ion in the pH range 2.0–11.0 in 0.15 mol/LNaCl ionic medium, at 310 K were taken in the wavelength range 250–450 nm. Metal concentration was held constant (2.5 × 10^−5^ mol/L). The pH of all solution was adjusted by the addition of 0.1 mol/LHCl or 0.1 mol/LNaOH. Solutions were left for 0.5 h before scanning. Another set of spectrophotometric data was performed as spectrophotometric titration. The solution from the titration cell was pumped after each addition of alkali to spectrophotometer flow through 10 mm quartz cuvette using peristaltic pump.

#### 3.3.3. Spectrofluorimetric Measurements

The excitation and emission spectra were taken for either levofloxacin or ofloxacin (2.0 mmol/L) with and without presence of copper ion. Aliquots of 0.1 mL of standard solution of either levofloxacin or ofloxacin were transferred into 10 mL volumetric flask, using Ependorf pipette and in each flask 2 mL of phosphate buffer (pH 7.40) and different volumes (0–0.3 mL) of copper ion solution (10 mmol/L)) were added. After dilution to the mark with water and thorough mixing, the fluorescence intensity of each spectrum was measured in wavelength range 400–650 nm with excitation at 292 nm.

#### 3.3.4. ESI-MS Measurements

For ESI-MS measurements FQ-metal solutions were prepared in the FQ to metal ion concentration ratio 2:1 at the pH 4.5 adjusted with ammonium formate buffer. Concentration of fluoroquinolone in solutions was 2 × 10^−4^ mol/L. The ESI–source parameters were as follows: source voltage 4.7 kV, capillary voltage 23 V, tube lens voltage 90 V, capillary temperature 220 °C, sheath gas flow (N_2_) 32 (arbitrary units). ESI-MS spectra were acquired by full range acquisition of *m/z* 200–2000. The normalized collision energy of the CID cell was set at 8–20 eV.

#### 3.3.5. NMR Measurements

All ^1^H-NMR spectra were recorded in DMSO-*d*_6_ and in D_2_O solutions. Typical conditions for ^1^H-NMR measurements were: spectral width 3,500 Hz, pulse delay time 1 s, no of scans 72. Chemical shifts (*δ*, ppm) were obtained for 3-trimethylsilylpropionic acid-d_4_ sodium salt as an internal standard (δ = 0.000 ppm).

### 3.4. Data Treatment

The species formed in the studied systems were characterized by the general equilibrium:

*pM*^2+^ + *qFQ*^− ^+ *rH*^+ ^↔ [*M_p_FQ_q_H_r_*]^(2*p*+*q*−*r*)^ (13)

and the corresponding constants are given by:

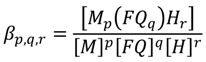
(14)
where FQ^−^ is the deprotonated molecule of the ligand. Fully protonated fluoroquinolone is donated as H_2_FQ^+^. The concentration stability constants of complexes *β_p,q,r_* were calculated with the aid of the suite of computer programs Hyperquad2006 [17]. In Hyperquad calculations the identity and stability of complexes which give the best fit to the experimental data, were determinated by minimizing the error squares sum of the potentials, *U: U = ∑w_i_(E_obs_ − E_calc_)^2^* where *w_i_* represents a statistical weight assigned to each point of titration curve, *E_obs_* and *E_calc_* refer to the measured potential of the cell and the calculated one assuming the specific model and trial constants, respectively. Quality of fit was judged by usual statistical parameters: Pearsons test, *χ^2^*, standard deviation in potential residuals, *s*, and the difference between experimentally determined and calculated standard EMF of the cell. If this difference was higher than 1 mV the titration was discarded. The spectrophotometric data were evaluated with Hyperquad2006 and pHab2006 programs. The composition, stability and molar absorptivities, *ε_p,q,r_* of complexes were determinated by minimizing the sum, S, defined as: S = *∑(A_obs_ − A_calc_)^2^* where *A_obs_* and *A_calc_* refer to measured absorbance and that calculated according to equation: *A_calc_* = *∑ β_p,q,r_[M]^p^ [FQ]^q^ [H]^r^ ε_p,q,r_*. Acceptance criteria for each particular model were: S lower than 1.0 × 10^−2^ and standard deviation of the fit of the spectrum (SD) less than 0.08 units. For Hyperquad calculations the spectra were digitized at 2 nm intervals. The final model obtained in Hyperquad calculations was optimized.

### 3.5. The Human Blood Plasma Model and Speciation Calculation

In developing the computer model of blood plasma, we updated the May *et al.* model of blood plasma and constructed a model including nine metals, 43 ligands and over 6,100 complexes [5]. The free concentration of all metal ions (component) was calculated by solving a mass balance equation written for each component:

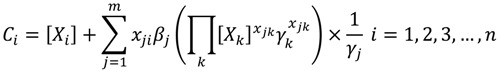
(15)
where *C_i_* is the total analytical concentration of component *i*, [*X_i_*] the concentration of free component *i*, and *X_ji_* the stoichiometric factor of component *i* in species *S_j_*. The species *S_j_* is formed from the components A, B, C… according to equations:

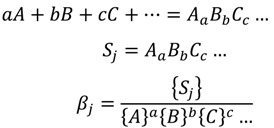
(16)
where {} denotes activity, and a,b,c… are stoichiometric factors of components A, B, C in species *S_j_*, respectively, and *β_j_* is the stability constant of *S_j_*.

Total concentrations of all components were taken from published papers and Geigy tables [53,54,55,56,57,58,59,60,61,62,63]. The stability constants were extracted from the literature and were necessary adjusted to 310 K and ionic strength of 0.15 mol/L [64,65,66]. The equilibrium constants not available in the literature were measured in this work or estimated using LFER approach. A complete list of complexes of components and constants was described in detail in our previous work [16].

## 4. Conclusions

Computer simulations are a useful way of predicting the effect of various exogenous ligands on the biodistribution of essential metal ions in biological fluids and tissues, particularly in human blood plasma. Fluoroquinolones affect to a significant degree the distribution of LMW species of the essential metal ions Cu and Zn and the non-essential one Ni. The effect of fluoroquinolones originates from their pronounced tendency chelate aforementioned metal ions and compete with their major binders in human plasma-amino acids. The quantitative evaluation of the mobilizing effect demonstrates that fluoroquinolones are safe regarding their ability to disturb LMW complexation equilibria in blood plasma and consequently change the metabolism of Cu, Ni and Zn ions. Thus, the amino-acid pool is metal protective in plasma and as computer simulation results show, quinolones cannot significantly disturb these equilibria under normal concentration conditions. However, if such conditions are (temporarily) changed, for example, after intravenous infusion of the drug the metal-quinolone interactions may become noticeable and consequently metal mobilization may take place. The observed effect emphasizes the need to take into the consideration not only free quinolones, but also metallo-quinolone complexes in any explanation of their antibacterial action.
